# Psychological characteristics of the patients with juvenile idiopathic arthritis (JIA) of various age groups

**DOI:** 10.1186/1546-0096-9-S1-P123

**Published:** 2011-09-14

**Authors:** NY Stepanenko

**Affiliations:** 1 Rheumatology Research Institute RAMS, Moscow, Russian Federation

## Objective

Detection of psychological characteristics of the children with JIA of different age groups.

## Materials

155 patients with JIA aged 4-17 years. Group 1 – aged 4-7 years (15 people), Group 2 – aged 8-12 years (40 people), Group 3 – aged 13-17 years (100 people).

## Methods

Family pattern (Karen Mahover) (Groups 1,2,3), House-Tree-Person (John N. Buck) (Group 1), Scale of Tailor by Spilberg-Hanin (Group 3), the Lüscher color test (Groups 2,3), Junior–Senior High School Personality Questionnaire by Cattell (HSPQ – teenager version) (Group 3), Picture of a non-existent animal (M.Z. Dukarevich) (Groups 2,3), Cattell’s methods for junior personality psychology study (Group 2).

## Results

### Group 1

fears (73%), communicative disorders (67%), high level of anxiety (40%), aggression (20%), low level of social adaptation (13%).

### Group 2

high level of anxiety (65%), communicative disorders (55%), fears (42%), increased level of psychoemotional stress (35%), aggression (25%), tendency toward depression (25%), low level of social adaptation (12%) and appearance complexes (12%).

### Group 3

communicative disorders (69%), high level of anxiety (58%), psychoemotional stress (37%), fears (34%), low level of social adaptation (24%), tendency toward depression (24%), appearance complexes (22%), aggression (20%).

## Conclusion

The studied groups showed the same levels of communicative disorders and aggression. Age increase was followed by the decrease in number of patients with fear; and by increase in number of patients with low level of social adaptation, the percentage of appearance complexes and psychoemotional stress appeared and was increasing. The defined characteristics should be considered during the individual work with the patients.

